# Computational affinity maturation of camelid single-domain intrabodies against the nonamyloid component of alpha-synuclein

**DOI:** 10.1038/s41598-018-35464-7

**Published:** 2018-12-04

**Authors:** Sai Pooja Mahajan, Bunyarit Meksiriporn, Dujduan Waraho-Zhmayev, Kevin B. Weyant, Ilkay Kocer, David C. Butler, Anne Messer, Fernando A. Escobedo, Matthew P. DeLisa

**Affiliations:** 1 Robert F. Smith School of Chemical and Biomolecular Engineering, Cornell University, Ithaca, NY 14853 USA; 2 Nancy E. and Peter C. Meinig School of Biomedical Engineering, Cornell University, Ithaca, NY 14853 USA; 3 Biological Engineering Program, Faculty of Engineering, King Mongkut’s University of Technology Thonburi, Bangkok, Thailand; 4 Neural Stem Cell Institute, Rensselaer, NY 12144 USA; 5 Department of Biomedical Sciences, University at Albany, Albany, NY 12208 USA

## Abstract

Improving the affinity of protein-protein interactions is a challenging problem that is particularly important in the development of antibodies for diagnostic and clinical use. Here, we used structure-based computational methods to optimize the binding affinity of V_H_NAC1, a single-domain intracellular antibody (intrabody) from the camelid family that was selected for its specific binding to the nonamyloid component (NAC) of human α-synuclein (α-syn), a natively disordered protein, implicated in the pathogenesis of Parkinson’s disease (PD) and related neurological disorders. Specifically, we performed *ab initio* modeling that revealed several possible modes of V_H_NAC1 binding to the NAC region of α-syn as well as mutations that potentially enhance the affinity between these interacting proteins. While our initial design strategy did not lead to improved affinity, it ultimately guided us towards a model that aligned more closely with experimental observations, revealing a key residue on the paratope and the participation of H4 loop residues in binding, as well as confirming the importance of electrostatic interactions. The binding activity of the best intrabody mutant, which involved just a single amino acid mutation compared to parental V_H_NAC1, was significantly enhanced primarily through a large increase in association rate. Our results indicate that structure-based computational design can be used to successfully improve the affinity of antibodies against natively disordered and weakly immunogenic antigens such as α-syn, even in cases such as ours where crystal structures are unavailable.

## Introduction

α-Synuclein (α-syn) is a presynaptic neuronal protein that is pathologically linked to a number of neurodegenerative disorders collectively known as synucleinopathies, including Parkinson’s disease (PD), dementia with Lewy bodies (DLB) and multiple system atrophy (MSA)^1–4^. While the exact ways that α-syn contributes to PD pathogenesis remain unclear, it is generally believed that aberrant soluble oligomeric α-syn conformations, termed protofibrils, are the toxic species that disrupt cellular homeostasis and cause neuronal death. Because α-syn may play a central role in pathogenesis, reducing intracellular levels to prevent the abnormal misfolding, aggregation, and toxicity in vulnerable cells may serve as a potential therapeutic strategy for slowing the progression of PD and other synucleinopathies where misfolded proteins and subsequent protein aggregation appears to be an underlying factor^4^.

To achieve such an outcome, intracellular antibodies (intrabodies) have been proposed as a strategy for targeting and/or neutralizing different aberrant α-syn species^5–8^. Intrabodies are antibody fragments that have been engineered to be expressed intracellularly where they bind their cognate target antigens, and have shown promise in infectious diseases^9,10^ and cancer^11,12^. Intrabodies have also been used to target molecular features of protein misfolding and aggregation in Huntington’s disease^13–15^, with follow-on studies providing *in vivo* proof-of-concept of the protective effects of such intrabodies^16–18^. More recently, intrabodies have been developed that have affinity for different conformations of α-syn (*e.g*., monomeric, oligomeric and fibrillar)^19–21^ or specific residues of α-syn (*e.g*., C terminal region)^22–24^. One of these, the single-chain Fv (scFv) intrabody NAC32 that is specific for amino acids 53–87 of α-syn comprising part of the nonamyloid component (NAC) region, was found to reduce the toxicity caused by the A53T mutant of α-syn in cultured cells^22^.

Unfortunately, it remains a significant challenge to isolate intrabodies that exhibit sufficient stability and binding affinity when expressed in living cells, specifically in the reducing environment of the cytoplasm. Regarding intracellular stability, only a small fraction of intrabodies are intrinsically soluble in the cytoplasm. Indeed, many promising intrabodies suffer from poor cytoplasmic solubility^25^, and are prone to misfolding and aggregation due to the redox potential and macromolecular crowding of the intracellular environment^26–28^. Because solubility remains a difficult property to predict, library-based strategies are currently the most effective tool for identifying solubility-enhanced intrabody variants^29–31^. Regarding affinity for antigen, suboptimal affinity may allow kinetic escape and accumulation of the target in a manner that prevents phenotypic inhibition or knockout. For example, an initial panel of eight huntingtin (htt)-specific scFv clones isolated from a synthetic, non-immune library using yeast surface display, was found to be completely inactive in preventing aggregation of htt in a yeast model of HD^32^. The affinity of the hits (in the micromolar range) was deemed to be insufficient for biological activity. In support of this notion, a tighter binding clone (*K*_d_ ≈ 30 nM) was isolated by affinity maturation using yeast display and found to efficiently block htt aggregation in cultured mammalian cells. However, while this finding illuminates the importance of strong binding affinity for intrabody performance, the process by which affinity was improved was labor intensive and time consuming, requiring construction of two combinatorial antibody libraries and a total of eight rounds of fluorescence-activated cell sorting (FACS)-based library screening. Likewise, we recently employed a directed evolution strategy to enhance the affinity of NAC32 and, while successful, it required multiple labor-intensive rounds of library construction and selection to yield a clone exhibiting a modest ~8-fold increase in binding affinity^31^.

To overcome some of the shortcomings associated with experimental affinity maturation, structure-based computational techniques have emerged as an important complement for achieving high-affinity binding. The advantage of computational techniques stems from their ability to screen optimal designs within a virtual library containing ~10^40^ sequences in just a few days, which is in contrast to experimental library search strategies (*e.g*., directed evolution) that interrogate ~10^10^ sequences per experiment over a timeframe of weeks to months. Computational strategies take advantage of algorithms such as Monte Carlo-based searches^33^ that permit large numbers of residue types and conformations to be screened *in silico* using fast evaluations of energetic properties. This is made possible by the compatibility of exhaustive search algorithms with the highest-quality methods for energy evaluations, especially the treatment of the solvent and electrostatic interactions, such as numerical solution of the Poisson-Boltzmann equation^34^. Indeed, progress in computing performance and force-field parameterization has made it possible to efficiently perform *in silico* affinity maturation whereby accurate biophysical models are leveraged to effectively guide the identification of antibody residues and motifs that maximize affinity for a target antigen epitope. Such approaches have been used by several groups to design mutations in antibodies that significantly improve their affinity towards antigen^35–41^.

Inspired by these earlier efforts, here we describe a strategy for computational affinity maturation of V_H_NAC1, a recombinant single-domain camelid antibody (VHH) specific for the NAC domain of α-syn. A coherent picture of the interaction between V_H_NAC1 and NAC was constructed using a combination of advanced molecular dynamics (MD) techniques (*e.g*., replica exchange molecular dynamics (REMD)^42^, umbrella sampling (US)^43^, and weighted histogram method (WHAM)^44^) together with numerical techniques (*e.g*., finite difference form of Poisson-Boltzmann equation (PBEQ)). This undertaking was non-trivial because, unlike the earlier *in silico* efforts discussed above, high-resolution structures for V_H_NAC1, the NAC antigen, and the V_H_NAC1-NAC complex do not exist. Moreover, we had to resolve the issue of the multitude of conformations that can be adopted by α-syn. Importantly, our *ab initio* modeling results allowed us to propose: (i) possible conformations of the V_H_NAC1 binding region within the NAC domain; (ii) possible modes of V_H_NAC1 binding to the NAC region; and (iii) mutations that enhance V_H_NAC1 binding to NAC, specifically by alleviating electrostatically suboptimal contacts at the binding interface. Unexpectedly, a single point mutation (N77D) was sufficient to enhance the affinity of V_H_NAC1 by more than an order of magnitude, with the resulting N77D mutant exhibiting high nanomolar binding affinity as confirmed by surface plasmon resonance (SPR) experiments. The improved affinity of this mutant was achieved primarily through an order-of-magnitude increase in association rate. Our results demonstrate that structure-based computational design can be used to significantly improve the affinity of intracellular antibodies even when crystal structures are not available.

## Results

### Selection of a VHH intrabody against NAC domain

Human α-syn is a 140-residue intrinsically disordered protein of unknown function that adopts different conformational forms upon interacting with other biomolecules^45,46^. The protein has an amphipathic N-terminus and acidic C-terminus that are separated by a central hydrophobic domain known as the NAC region (amino acids 61–95; Supplementary Fig. 1a). The NAC domain is prone to aggregation, forming beta-sheets by self-association, and is considered to be key for the assembly of α-syn into fibrils^47,48^. Importantly, deletion of the amino acids 71–82 from the NAC domain abrogates aggregation and significantly reduces toxicity in a transgenic Drosophila model of the disorder^49^. Taken together, these findings point to the NAC domain as an attractive target for intrabody development.

**Figure 1:**
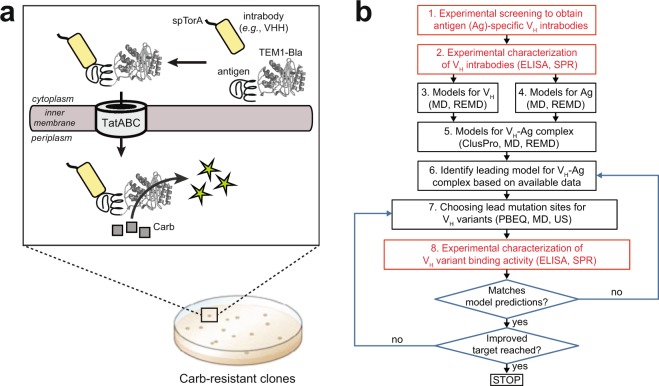
Identifying recombinant intrabodies using FLI-TRAP. (**a**) Schematic of engineered genetic selection for co-translocation of cognate intrabody-antigen pairs via the Tat translocase, TatABC. Here, an immune library of NAC-focused VHH genes was fused to a Tat signal peptide, namely spTorA, and co-expressed with a chimera between the antigen α-syn(A53T) and the reporter enzyme TEM1-Bla. By demanding cell growth on selective amounts of β-lactam antibiotics (*e.g*., Carb), intracellularly stable VHH clones that bind α-syn(A53T) can be readily isolated from the library. (**b**) Iterative workflow based on estimates from experiments for updating computational models and making new predictions.

To generate an initial VHH intrabody against the NAC region of α-syn, we used a previously reported genetic selection, termed FLI-TRAP (functional ligand-binding identification by Tat-based recognition of associating proteins), for isolating binding proteins from combinatorial libraries^50^. This strategy leverages the inbuilt ability of the twin-arginine translocation (Tat) system to efficiently localize non-covalently assembled protein complexes to the periplasm of *Escherichia coli* cells^51^. The assay involves two engineered fusion proteins: (i) a Tat signal peptide (*e.g*., spTorA) fused to the N-terminus of a designer binding protein (*e.g*., scFv, VHH), and (ii) the corresponding antigen (*e.g*., α-syn) fused to the reporter enzyme, TEM-1 β-lactamase (Bla) (Fig. 1a). Co-translocation of the antigen-Bla chimera to the periplasm by the spTorA-intrabody fusion enables semi-quantitative, high-throughput selection of pairwise interacting proteins that interact with sufficiently high affinity in the cytoplasm. Thus, bacterial resistance to antibiotic can be used as a phenotypic readout for the discovery of antigen-specific intrabodies that are optimized for cytoplasmic expression. Importantly, we previously adapted this assay for identifying and optimizing scFv intrabodies against the A53T mutant of α-syn^31,50^, a highly toxic version of α-syn^22^.

Here, a combinatorial library of NAC-focused VHH genes, which was prepared from B cells obtained following alpaca immunization with a peptide comprising the NAC region of α-syn (Supplementary Fig. 1b), was cloned into the FLI-TRAP plasmid as a fusion to the spTorA signal peptide (Fig. 1a). To isolate NAC-specific clones from this library, the spTorA-VHH library was co-expressed in *E. coli* cells along with the α-syn(A53T)-Bla fusion, resulting in the outgrowth of bacterial colonies that were resistant to carbenicillin (Carb). Six colonies were chosen for further analysis; however, following sequencing only two clones were confirmed to be full-length, unique VHH sequences. These two VHH clones exhibited Carb resistance that was greater than that conferred by a non-specific green fluorescent protein (GFP)-binding VHH (cAbGFP4)^52^ or a randomly chosen non-binding VHH clone, V_H_NB10, and on par with that conferred by the positive control NAC32 (Supplementary Fig. 2a), an scFv intrabody that is specific for the NAC region of α-syn^22^. Only one of these clones, designated V_H_NAC1, was confirmed to bind immobilized α-syn(A53T) above background in ELISA experiments (Supplementary Fig. 2b). However, while the binding activity of V_H_NAC1 was clearly above the non-binding V_H_NB10 clone and non-specific cAbGFP4, it was notably lower than that observed for NAC32 despite the two proteins being expressed at relatively similar levels (Supplementary Fig. 2b). Hence, we concluded that V_H_NAC1 was a suitable candidate for affinity improvement using computational methods. A summary of our approach is provided as a flow diagram (Fig. 1b) whose feedback loops can be seen as an iterative approach to predict the most stable complexes, wherein the prior estimates are refined (into posterior estimates) in light of new information.

### Atomistic model of V_H_NAC1 and its complex with NAC peptide

**Figure 2:**
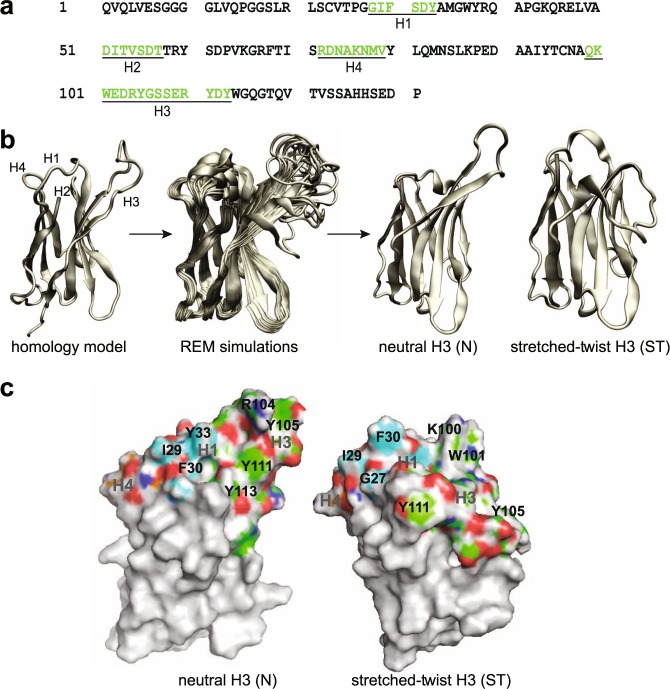
A tertiary structural model for V_H_NAC1 starting from primary sequence. (**a**) Amino acid sequence of camelid VHH intrabody V_H_NAC1 specific for NAC domain of α-syn. Hypervariable regions H1-H4 are labeled using Kabat scheme^76^ and loop definitions as described^55^. (**b**) Enhanced conformational sampling to obtain top two conformations: neutral H3 (N) and stretched-twist H3 (ST) from homology model. (**c**) Binding surface formed by hypervariable loops – colored using heteroatom scheme – main chain of H1 is cyan, H2 is yellow, H3 is green and H4 is orange.

The starting structure for V_H_NAC1 was generated using the homology model engine of Swiss-Model^53^ and was based on the solved crystal structure for an anti-β2 adrenergic receptor nanobody (PDB: 3P0G)^54^. While homology models (and other knowledge-based methods) are very efficient at predicting the framework regions due to high sequence similarity, these methods can produce loop conformations with considerable deviations from the correct or native conformations due to high sequence and length variability in the variable regions of VHHs^55^. Hence, we performed all atomistic REMD simulations in implicit solvent to obtain the equilibrium conformations of the binding loops of V_H_NAC1. All simulations employed GROMACS^56^ using the CHARMM27 force field in implicit solvent^57^. All residues except those in the hypervariable loops H1, H2, H3, and H4 were backbone-restrained. This technique was previously used to successfully predict the conformation of VHH loops^58,59^. Furthermore, we retained all of the V_H_NAC1 residues (Fig. 2a) for the simulations as we found that the “reduced model” used in the earlier studies was only suitable for modeling shorter H3 loops (<12 amino acids). For longer H3 sequences, the loops may form contacts with many framework residues not included in the reduced model. By using REMD simulations for enhanced conformational sampling of the loop regions, we obtained two main structures for the binding loops. Notably, in the two main structures, the H3 loop exhibits either a neutral (N; 42% of all conformations sampled) or a stretched-twist (ST; 22% of all conformations sampled) conformation (Fig. 2b). Both structures were retained for further simulations and analyses. The N and ST conformations lead to two very distinct binding surfaces (Fig. 2c). In both conformations, there is a clustering of hydrophobic residues at the interface of H1 and H3 loops. This is a preliminary indication of the possible location of the paratope on V_H_NAC1. Both conformations exhibit groove-like cavities, as one would expect for proteins that bind to peptides or disordered proteins.

To computationally investigate V_H_NAC1 antigen binding, a model for the NAC domain was needed. While it is generally accepted that α-syn is largely unfolded in solution *in vitro* and *in vivo*^45^, the possibility that transient local structural motifs/features are present has not been ruled out. In fact, multiple studies have reported the presence of transient α-helical character in the NAC region^46,60^. Consequently, we considered two models for the NAC region: (i) a 19-residue α-helical peptide (residues 61–79) observed with solution NMR spectroscopy of α-syn in the micelle-bound form (PDB: 1XQ8)^61^; and (ii) a 13-residue random coil peptide generated by piecing together the amino acids (residues 66–78) one-by-one (see Supplementary Fig. 1b for sequences). We chose these two models to account for the propensity of α-syn to adopt different conformations^45,46^ and the possibility that a state of dynamic equilibrium exists between these conformations^46,60^, which could be important for specific recognition by our VHH intrabody.

**Figure 3:**
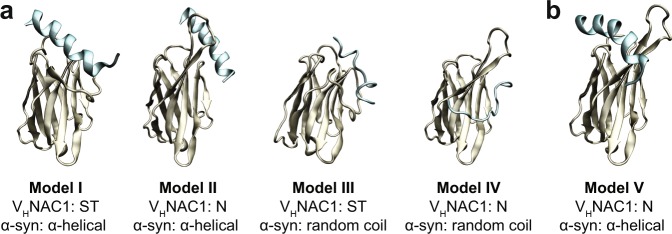
Models of V_H_NAC1 in complex with NAC peptide. (**a**) Top four models obtained from simulations of the V_H_NAC1-NAC complex and (**b**) a fifth model that was chosen based on SPR results. Models I, II and V were obtained for a 19-residue α-helical peptide from the NAC domain (amino acids 61–79) while Models III and IV were obtained for a 13-residue random coil peptide from the NAC domain (amino acids 66–78). NAC peptides are shown in cyan.

To generate a model of the V_H_NAC1-NAC complex, docking was performed using ClusPro^62–65^. Overall, 41 initial structures against the 19-residue α-helical (23 structures) and 13-residue random coil (18 structures) NAC peptides were obtained from four docking runs in ClusPro: (i) V_H_NAC1 (N conformation) with 19-mer α-helical peptide; (ii) V_H_NAC1 (N conformation) with 13-mer random coil peptide; (iii) V_H_NAC1 (ST conformation) with 19-mer; (iv) V_H_NAC1 (ST conformation) with 13-mer (Supplementary Fig. 3). Energy minimization and equilibration MD runs were performed for all structures. A shorter, 13-residue peptide was used for the random coil conformation of the NAC region since none of the docked structures obtained from ClusPro for a longer 19-residue random coil were stable in energy minimization and/or equilibration MD simulations. This was attributed to the unwieldy nature of the 19-mer random coil peptide, which was much longer than the α-helical peptide model having the same number of residues. After energy minimization and equilibration at 300 K for all structures, four separate temperature-based-REMD simulations were performed for each of the different types of complex described above for enhanced conformational sampling and, ultimately, to rank structures from the most stable to the least stable at the temperature of interest (Fig. 3a shows the top four models and Supplementary Fig. 4 shows all the models obtained). To characterize the models, we obtained the average energy of interaction between the V_H_NAC1 and NAC antigen for each model. The energy of interaction was calculated separately for the van der Waals type interactions and Coulombic interactions (Supplementary Fig. 5a).

### Enhancing the energy of binding between V_H_NAC1 and disordered NAC

As a first pass at affinity maturation (Fig. 1b, steps 6 and 7), we focused on the disordered NAC antigen because this is the more likely conformation *in vivo*^45^. Of the models involving disordered NAC antigen, Model III involving the ST V_H_NAC1 structure was initially chosen to make further predictions. There are multiple factors to consider when aiming to improve binding (*i.e*., implement step 7 in Fig. 1b) such as: (i) the considerable loss of conformational entropy of the NAC peptide upon binding; (ii) some compensation of the decrease in conformational entropy by the gain in entropy of water molecules upon the desolvation of the hydrophobic residues in the NAC peptide; and perhaps most importantly (iii) the gain in enthalpy due to new contacts formed at the interface, which contributes favorably to free-energy of binding. The first issue is especially significant for a disordered antigen due to the significant loss of entropy upon creating a “stabilizing/ordered” contact upon binding. Hence, we followed a more cautious strategy as described below.

The NAC region is primarily hydrophobic. However, the core hydrophobic residues (66-VGGAVVTGVTAVA-78) are flanked on both sides by hydrophilic residues (57-EKTKEQVTN-65 and 79-QKTVE-83). For the Model III complex, the hydrophobic core fits into a paratope on V_H_NAC1 composed primarily of hydrophobic residues. One strategy to improve binding would be to optimize the electrostatic complementarity between the flanking hydrophilic sequences and V_H_NAC1. This strategy would effectively keep the hydrophobic core of the V_H_NAC1-NAC binding interface intact while optimizing the interactions at the rim. Moreover, computational alanine scanning and salt-bridge analysis indicate that interfaces in intrinsically disordered protein complexes are highly complementary with respect to electrostatics, more so than interfaces of globular proteins^66^.

To understand the effect of these polar/hydrophilic residues, we ran umbrella sampling (US) simulations to obtain an estimate for the potential of mean force (PMF) as a function of separation between V_H_NAC1 and the NAC region for two versions of the antigen: (i) the 13-residue hydrophobic random coil peptide (residues 66–78); and (ii) a 24-residue peptide (residues 57–80) (Supplementary Fig. 1b). Sample simulations were run for Model III. For this model, US simulations revealed a less favorable complex with the 24-residue peptide (Supplementary Fig. 5b). This implied that the electrostatic interactions were significant and potentially suboptimal, thereby leading to a less favorable PMF (~10 kT/mol) for the formation of this complex. Hence, based on these PMF estimates, another straightforward approach for affinity maturation is to optimize the electrostatic interactions at the binding interface, which could potentially lead to a 10 kT/mol (4 kcal/mol) improvement in binding free-energy.

### Improving electrostatic complementarity at the rim of the binding interface

**Figure 4:**
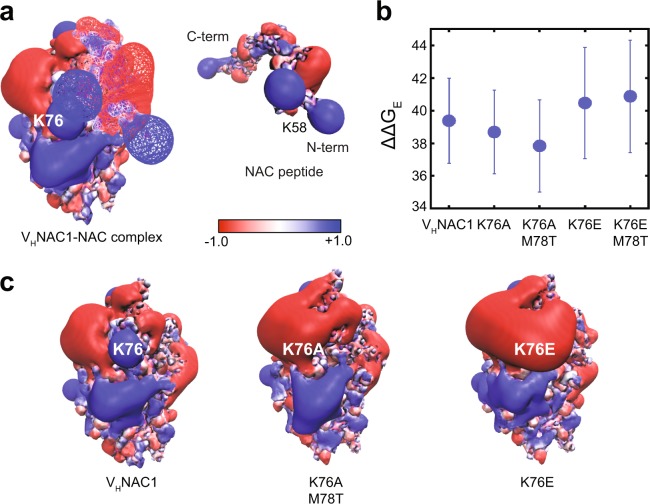
Electrostatic potential surfaces for Model III of V_H_NAC1-NAC complex. (**a**) Electrostatic potential surfaces for Model III of the complex between V_H_NAC1 and NAC peptide (left) and for the NAC peptide by itself (right). (**b**) The electrostatic free energy (FE) of binding calculated using PBEQ for V_H_NAC1 and the K76A, K76A/M78T, K76E and K76E/M78T mutants. (**c**) Electrostatic potential of V_H_NAC1 and the K76A/M78T and K76E mutants. For the complex, the V_H_NAC1 is depicted as a solid surface while the NAC peptide is depicted as a wire-mesh. For the NAC peptide, the surface is shown as a solid surface. Blue indicates an electrostatic potential of 1 kT/e and red indicates an electrostatic potential of −1 kT/e.

To analyze electrostatic interactions, we used the finite-difference form of the PBEQ implemented in DelPhi software^67–69^. We obtained the PBEQ surface for V_H_NAC1 and NAC antigen corresponding to Model III, which revealed two primary residues on V_H_NAC1 that were interacting unfavorably with the antigen. These residues were lysine at position 76 (K76) and lysine at position 99 (K99). The unfavorable energetics were most likely due to the proximity of V_H_NAC1 residue K76 to NAC residues K58 and K60 (Fig. 4a). Similarly, V_H_NAC1 residue K99 is proximal to NAC residue K80. Since PBEQ is a numerical calculation (averaged over multiple snapshots of the VHH, antigen, and the complex collected over a 1-ns long MD simulation of the equilibrated structures), it is computationally much faster than US simulation (~1000 ns of cumulative simulation time), and thus was used as a first step for scanning multiple mutants for improved affinity. We thus proposed four V_H_NAC1 variants – K76A, K76E, K76A/M78T, and K76E/M78T – each of which was aimed at alleviating electrostatic repulsion at the binding interface for Model III. Following equilibration simulations and PBEQ analysis of the equilibrated structures, mutants K76A and K76A/M78T were found to have an improved electrostatic free energy of binding over parental V_H_NAC1 while mutants K76E and K76E/M78T exhibited the opposite behavior (Fig. 4b and c). Subsequent US simulations for these mutants confirmed K76A/M78T as a suitable candidate for enhanced affinity (Supplementary Fig. 5c).

To test these findings, we expressed and purified V_H_NAC1 and the K76A/M78T and K76E mutants (Fig. 1b, step 8), and subjected each to SPR analysis to determine the rates of association, dissociation and the dissociation constant. Counter to our expectations, we observed a considerable weakening of binding for the variants with respect to parental V_H_NAC1, with the observed trend for binding affinity opposite to that predicted in simulations (Table 1). Interestingly, we did notice that changing the salt concentration of the buffer led to a significant increase in rates of association (*k*_on_), and thus stronger affinity, with *k*_on_ values in the following order: V_H_NAC1 > K76A/M78T > K76E. Taken together, while the designed mutations did not improve the affinity, they did reveal the importance of long-range electrostatic interactions.

**Table 1:** SPR results for V_H_NAC1 and mutants.

VHH construct	[NaCl] (mM)	*K*_d_ (M)	χ^2^
V_H_NAC1	150	3.22E-06	1.18
K76A/M78T	150	9.23E-04	0.42
K76E	150	1.37E-03	1.29
V_H_NAC1	75	2.43E-07	2.11
K76A/M78T	75	2.96E-06	2.90
N77D	150	6.64E-08	2.35

### Improving electrostatic complementarity at the binding interface

**Figure 5:**
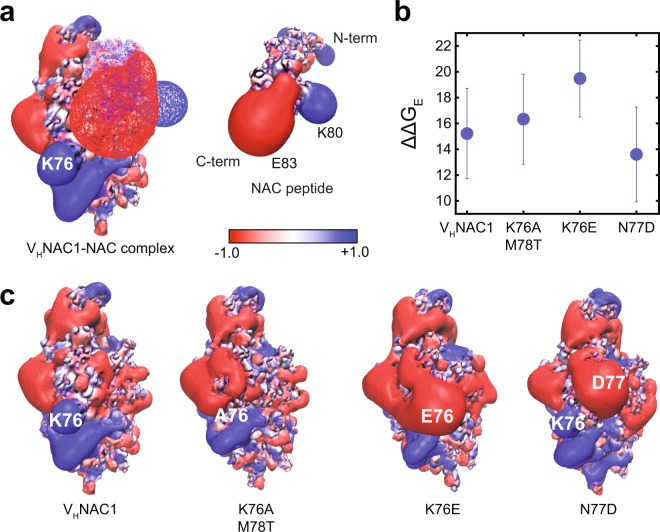
Electrostatic potential surfaces for Model V of V_H_NAC1-NAC complex. (**a**) Electrostatic potential surfaces for Model V of the complex between V_H_NAC1 and NAC peptide (left) and for the NAC peptide by itself (right). (**b**) The electrostatic free energy (FE) of binding calculated using PBEQ for V_H_NAC1 and the K76A/M78T, K76E, and N77D mutants. (**c**) Electrostatic potential of V_H_NAC1 and the K76A/M78T, K76E, and N77D mutants. For the complex, the V_H_NAC1 is depicted as a solid surface while the NAC peptide is depicted as a wire-mesh. For the NAC peptide, the surface is shown as a solid surface. Blue indicates an electrostatic potential of 1 kT/e and red indicates an electrostatic potential of −1 kT/e.

Our results above suggested that the positively charged K76 residue was important for binding but the rate of association weakened upon changing the residue from positive to neutral to negative. To develop a better model for the complex (Fig. 1b, iterating between steps 6–8), two possibilities were considered: (i) K76 could be important for long-range interaction with the negatively charged C-terminus of α-syn; and/or (ii) K76 could be interacting with certain negatively charged residues in the NAC region. We scanned the remaining models (Models I, II, and IV) and found that Model I as well as Model V, the fifth model selected for further analysis from the top models obtained from REMD simulations (Fig. 3b), satisfied these criteria. Incidentally, Model V was also the top ranked model for the V_H_NAC1 (N conformation) in complex with the 19-residue α-helical NAC peptide according to our ClusPro analysis (Supplementary Fig. 3a). Both models were tested using PBEQ calculations with results indicating that calculations based on Model V agreed more closely with the experimental results. In this model, the flanking C-terminal hydrophilic residues of the NAC peptide, but not the N-terminal ones, are in close proximity to residue K76 (Fig. 5a). These C-terminal residues are less positively charged, with residue E83 in this region of the NAC peptide interacting with K76 of V_H_NAC1. Moreover, in this model the negatively charged C-terminal residues of the NAC peptide are closer to K76 of V_H_NAC1 and may also influence long-range interactions. Hence, PBEQ calculations were performed for V_H_NAC1 and mutants K76A/M78T and K76E for Model V. From this analysis, we find that while the K76A/M78T double mutant has an electrostatic free energy of binding slightly worse than V_H_NAC1, the K76E mutant was observed to be considerably worse (Fig. 5b and c). The trend for *k*_on_ was in the order V_H_NAC1 > K76A/M78T > K76E, which agreed with the experimentally measured values for *k*_on_ estimated by SPR. This can be explained by the disruption of favorable interactions between A75 of the K76A/M78T double mutant and E83 of the NAC peptide, and introduction of repulsive interactions between E75 of the K76E mutant and E83 on the NAC peptide. The electrostatic surface generated from PBEQ analysis for V_H_NAC1 was also used to propose the mutation N77D, which introduces a new interaction between this residue in V_H_NAC1 and residue K80 in the C-terminal flanking region of NAC. It is apparent from the PBEQ surface that residues K76 and D76 of the N77D mutant will form a surface complementary to NAC residues E83 and K80, respectively (Fig. 5c), and this likely explains the more favorable electrostatic free energy of binding compared to V_H_NAC1 (Fig. 5b). Subsequently, US simulations were performed to obtain PMF of V_H_NAC1 and the N77D mutant for Model V. The PMF of binding for N77D was about 10 kT larger than that for V_H_NAC1, indicating stronger binding (Supplementary Fig. 5d).

To test these findings, we compared the α-syn(A53T)-binding activity of the N77D mutant with that of V_H_NAC1 (Fig. 1b, step 8). In agreement with our simulation results, the N77D mutant conferred higher resistance in the genetic selection assay and showed significantly stronger binding to immobilized α-syn(A53T) in ELISA experiments compared to V_H_NAC1 (Supplementary Fig. 2). When purified N77D mutant and V_H_NAC1 were each subjected to SPR analysis, we observed that N77D exhibited affinity for α-syn(A53T) that was significantly stronger than that measured for parental V_H_NAC1 (Table 1 and Supplementary Figs 6–8) and on par with the previously reported affinity (*K*_d_ = 46 nM) measured for NAC32^31^. Importantly, this large improvement in binding affinity was evident based on (i) approximate *K*_d_ values derived by plotting the plateau levels against α-syn(A53T) concentration and fitting the equilibrium binding responses (Supplementary Fig. 6) or (ii) *K*_d_ values obtained from a global fit of the data to a 1:1 Langmuir binding model using a simultaneous non-linear program (Table 1 and Supplementary Fig. 8). Dynamic light scattering (DLS) of the purified protein samples revealed a distinct peak, likely corresponding to monomeric VHH proteins (and perhaps also small aggregates or contaminants), for both V_H_NAC1 and N77D (Supplementary Fig. 6a). The DLS data also revealed a peak at >100 nm in both samples, but this peak corresponded to <0.1% of the mass. Hence, the majority of the VHH in each sample was non-aggregated, suggesting that the differences in affinity measured for V_H_NAC1 and N77D were unlikely to be the result of differences in micro-aggregation behavior. Rather, the affinity enhancement was almost entirely attributable to a large increase in the association rate, *k*_on_, for N77D compared to that of V_H_NAC1. Taken together, the trends observed for *k*_on_ in the SPR experiments (N77D > V_H_NAC1 > K76A/M78T > K76E) are indicative of a modulation of electrostatic interactions at the binding interface driven by computational design.

## Discussion

Here, we have demonstrated that it is possible to use structure-based computational design to significantly enhance the affinity of a single-domain camelid intrabody against the NAC domain of α-syn. Our work represented a significant departure from previous computational affinity maturation studies^35–38^ because no high-resolution structures for any of the components of our system – the intrabody, the antigen, or the intrabody-antigen complex – were available *a priori*. Instead, we used the amino acid sequence of the intrabody and its antigen to develop a model of binding *in silico*, and then used the model to predict affinity-enhancing mutations. Specifically, advanced MD techniques were used to obtain two unique equilibrium conformations of the binding loops of V_H_NAC1, one in which H3 exhibited a neutral conformation and another in which it adopted a stretched-twist conformation. For the antigen, we considered two models for the NAC region, a 19-residue α-helical peptide and a 13-residue random coil peptide. This was particularly important because if the α-syn molecule is in a state of dynamic equilibrium between multiple transient conformations^45,46,60^, binding might be specific to only one conformation. Alternatively, binding might result in arresting NAC in a specific conformation. Altogether, by considering two distinct V_H_NAC1 structures along with specific secondary conformations of the NAC peptide, we were able to generate a wide spectrum of possibilities for the final structure of the V_H_NAC1-NAC complex. In total, 41 such structures were generated and subsequently ranked from the most stable to the least stable as determined by MD simulations, which enabled enhanced conformational sampling for each complex.

Our approach is similar to ensemble dock approaches wherein ensembles of conformations are generated for one or both binding partners before using docking methods to predict the structure of the complex^70,71^. A distinct advantage that our methodology has over most ensemble docking methodologies is its effectiveness in sampling diverse conformations. For example, we are able to sample conformations as diverse as ST and N for loops H3 using REMD simulations in implicit solvent, which allows fast conformational sampling. Such diversity is difficult when ensembles are generated primarily in the neighborhood of initial conformations. This is especially important in the absence of a solved starting structure for V_H_NAC1.

From these models, electrostatic optimization methods were used to identify potential affinity-enhancing mutations with a particular focus on alleviating electrostatically suboptimal contacts at the binding interface. While our initial design strategy did not lead to improved affinity, it revealed a key residue on the paratope and the participation of H4 loop residues in binding, confirmed the importance of electrostatic interactions, and ultimately guided us towards a model that was better aligned with experimental observations. Notably, the final successful model for binding was comprised of the top model for V_H_NAC1 from REMD simulations (N conformation of H3 loop) and the top model for its complex with the α-helical NAC peptide predicted by ClusPro. This model led to the N77D design that was experimentally confirmed to bind antigen with greater affinity than its progenitor V_H_NAC1. These observations further boost confidence in the capacity of current modeling tools and techniques for generating experimentally relevant predictions.

A key advantage of computational design strategies such as the one described here and elsewhere^35–38^ is that, in principle, a small, experimentally tractable set of putative affinity-enhancing mutations can be identified. As a result, subsequent confirmation by site-directed mutagenesis or even a focused library approach can be carried out significantly faster than would be required for iterative random mutagenesis approaches. Moreover, for the design strategy to be considered robust, it should yield a considerable fraction of designs that are successful when tested experimentally. Along these lines, we designed six unique V_H_NAC1 variants each containing 1–3 mutations total, which is well within the range of experimental techniques for manual mutagenesis and affinity determination. Three of these designs (K76A/M78T, K76E and N77D) were produced recombinantly and evaluated for antigen binding by SPR, and one (N77D) exhibited affinity for α-syn(A53T) that was significantly increased compared to the parental V_H_NAC1 intrabody (a success hit rate of 33%). This enhancement could be attributed to an order-of-magnitude increase in the association rate. Altogether, the results described here represent an important step towards developing rational design strategies based on *ab initio* modeling and bottom-up design approaches that provide a deeper understanding of protein surfaces and interactions, and facilitate the design of mutations that modulate these interactions.

Even though the K76A/M78T and K76E designs were not successful, they proved to be very useful in refining the search methodology in a manner that guided us directly to the affinity-matured N77D design. Indeed, failed designs have proven incredibly useful in learning about deficiencies in the energy functions, search procedures, or other methodology in previous structure-guided computational design efforts. For example, Clark *et al*. reported an initial success hit rate of 12% (10 mutants with measured affinities better than wild-type out of 83 constructed mutants), but upon refining their protocol for predicting ΔΔ*G*s of binding using information from all constructed designs, they retroactively improved their success rate to 26% (9 mutants with increased affinity out of 35 total designs)^38^. Unsuccessful designs are also important reminders that computational design relies heavily on the accuracy of the structure; hence, the lack of a high-resolution structure for V_H_NAC1-NAC contributed to the difficulty in choosing an accurate model and making reliable predictions. However, structure alone is unlikely to be the sole factor given that the rather low 12% hit rate reported by Clark *et al*. involved computational predictions based on a moderate resolution (2.8 Å) crystal structure of the antibody-antigen complex^38^. Indeed, better treatments for entropic contributions, explicit water molecules, hydrogen bonding networks, and solvation effects, among others, are needed. With continued improvements in computational methodology, we anticipate that structure-based design will become an important tool for optimizing critical intrabody properties such as affinity, stability, and solubility.

## Materials and Methods

### Bacterial strains and plasmids

*E. coli* strain DH5α was used for plasmid cloning and library construction, strain MC4100 was used for genetic selection using FLI-TRAP^50^, and strain BL21(DE3) was used to express proteins for ELISA and SPR analysis. Cultures were typically grown in Luria Bertani (LB) medium supplemented with the appropriate antibiotic, and protein expression was induced with isopropyl β-D-1-thiogalactopyranoside (IPTG, 0.1 mM) or arabinose (0.2–1.0% w/v) depending on the plasmid used. Antibiotics were supplemented at the following concentrations: ampicillin (Amp, 100 μg/mL); chloramphenicol (Cm, 25 μg/mL); tetracycline (Tet, 10 µg/ml), and kanamycin (Kan, 50 μg/mL).

The immune-focused VHH library (available from Addgene as a synuclein phagemid library; https://www.addgene.org/pooled-library/messer-synuclein-vhh/) was generated previously by alpaca immunization with a fusion protein between biotin and a NAC-containing peptide derived from α-syn (amino acids 53–87; Supplementary Fig. 1b). Here, the immune VHH library was PCR amplified from the synuclein phagemid library and cloned in place of the gene encoding NAC32 in pDD18-spTorA-NAC32-FLAG^31^, yielding pDD18-spTorA-VHHlib-FLAG in which each VHH library gene was cloned as a fusion to the Tat-dependent spTorA signal peptide. For the NAC antigen, we used plasmid pDD322-TatABC::α-syn(A53T)-Bla, which encodes additional copies of the Tat machinery for efficient protein export and a fusion protein between the α-syn A53T mutant and TEM-1 Bla^31^. To express unfused versions of different binding proteins for ELISA and SPR analysis, we cloned genes encoding NAC32 scFv, cAbGFP4, V_H_NAC1 and the designed V_H_NAC1 mutants between the *Nde*I and *Not*I sites of plasmid pET-21a(+), which introduced a 6x -His tag to the C-terminus of each gene product. During the cloning procedure, a C-terminal FLAG tag was also introduced.

### Bacterial growth and library selection

For library selection, electrocompetent DH5α cells were transformed with the VHH library in plasmid pDD18-spTorA-VHHlib-FLAG. Clones containing library plasmids were recovered by selection on LB agar supplemented with Cm. The library, which was determined to contain ~1 × 10^6^ members, was miniprepped from DH5α. Next, electrocompetent MC4100 cells already harboring the pDD322-TatABC::α-syn(A53T)-Bla plasmid were transformed with library plasmid DNA and incubated at 37 °C for 1 h without any antibiotics. These transformed cells were then subcultured into fresh LB containing 25 µg/ml Cm and 10 µg/ml Tet to ensure that cells contained both plasmids and grown overnight. The next day, cells were spun down and normalized to OD_600_ ≈ 2.5 in fresh LB. 100-µL aliquots of serially diluted cells (including the dilution previously determined by spot plating) were then directly plated on LB agar supplemented 25–100 μg/ml Carb and 1.0% arabinose. Following incubation at 30 °C for ~48 h, hits were randomly picked and screened by spot plating to confirm Carb resistance. All hits deemed to be positive were then sequenced.

Spot plating of bacteria was performed using MC4100 cells carrying plasmid pDD18-spTorA-X-FLAG (where X = NAC32 scFv, cAbGFP4, V_H_NB10, V_H_NAC1 or one of the designed V_H_NAC1 mutants) and plasmid pDD322-TatABC::α-syn(A53T)-Bla. Briefly, cells were grown overnight at 37 °C in LB medium supplemented with appropriate antibiotics. The next day, β-lactam resistance was determined by spot plating 5 µL of serially-diluted overnight cells that had been normalized in fresh LB to OD_600_ ≈ 2.5 onto LB agar plates supplemented with varying amounts of Carb (10–100 μg/ml) or onto control plates supplemented with 25 μg/ml Cm. Following plating, bacteria were cultured at 30 °C for ~48 h.

### Western blot analysis and ELISA

Soluble whole cell lysates were prepared by pelleting 20–25 mL of induced cell culture and resuspending in 500 μL PBS to achieve normalized final OD_600_ ≈ 75. Resuspended cells were sonicated on ice and then spun down at 16,000 rcf for 20 min at 4 °C. The resulting supernatant was recovered as the soluble whole-cell lysate fraction. The resulting pellet was washed twice with 1 mL Tris-HCl (50 mM) with EDTA (1 mM) and resuspended in 500 μL PBS with 2% SDS. The resuspended pellets were boiled for 10 min and then centrifuged for 10 min at 16000 rcf. The supernatant was recovered as the insoluble fraction. Proteins in recovered fractions were electrophoretically separated using 12% SDS-polyacrylamide gels (BioRad), and then subjected to Western blot analysis according to standard protocols. Briefly, proteins were transferred onto polyvinylidene fluoride (PVDF) membranes, and intrabodies were detected by probing membranes with mouse anti-FLAG antibody conjugated to horseradish peroxidase (HRP) (Sigma-Aldrich).

To evaluate binding to α-syn, ELISA plates were coated with 1 μg/ul purified α-syn(A53T) peptide (Genway) in bicarbonate buffer overnight at 4 °C. Plates were then blocked with 5% non-fat milk in TBS for 2 h at room temperature and then washed using TBS supplemented with 0.1% Tween 20 (TBST). Whole cell lysate samples derived from BL21(DE3) cells expressing intrabodies from plasmid pET-21a were serially diluted in TBS with 1% BSA (TBS-BSA) and added to the plates (40 μL/well). Plates were incubated for 1 h at room temperature and then washed with TBST. After washing, plates were incubated with anti-FLAG antibody conjugated to HRP in TBS-BSA (50 μL/well) for 1 h at room temperature. After washing, plates were incubated with TMB HRP substrate (Thermo Fisher Scientific) for 10–20 min. The reaction was quenched with 2 M H_2_SO_4_, and absorbance in each well was measured at 450 nm.

### Protein purification

Intrabodies were purified from soluble whole cell lysates by Ni-NTA chromatography (Qiagen) using gravity columns according to standard protocols. Soluble lysates were incubated with the Ni-NTA resin for 1 h at 4 °C on a rotating platform. The lysates with the resin were poured into columns, until all the lysate and resin flowed into the column. The flow-through was poured again over the resin and allowed to flow-through again. The columns were washed four times with a buffer containing 20 mM NaH_2_PO_4_, 300 mM NaCl, and 90 mM imidazole (pH 7.5). Proteins were eluted using a buffer containing 20 mM NaH_2_PO_4_, 300 mM NaCl, and 250 mM imidazole (pH 7.5). After affinity purification, eluted proteins were concentrated followed by size exclusion chromatography (SEC). Briefly, 2.5 ml of concentrated protein was injected (1 ml at a time) into a Superdex 75 column (GE Healthcare) attached to an FPLC system (GE Healthcare) and samples were run at a flow-rate of 0.5 mL/min at 4 °C in buffer containing 15 mM Tris-HCl, 500 mM NaCl. The high salt concentration was used to prevent aggregation. The migration of intrabodies was monitored by absorbance at 280 nm, and 250 μL fractions were collected.

### Protein aggregation analysis using DLS

Analysis of protein aggregation using DLS was performed as described previously^72^. Briefly, purified VHH proteins were concentrated to >0.1 mg/ml and 400 μl was added to disposable plastic cuvettes (BrandTech Scientific) for DLS measurements using a Zetasizer Nano ZS (Malvern Instruments) at a backscatter angle of 173°. Analysis was performed using Malvern Zetasizer software version 7.10. Dispersant viscosity was approximated using the Zetasizer software dispersants manager for complex solvents. The measurement position and attenuator were set to automatic and the temperature was set to 4.0 °C. The protein analysis model was used with the display range set between 2 nm and 6,000 nm and thresholds set to their default values of 0.05 and 0.01, respectively. For each sample, quadruplicate measurements consisting of 20 runs of 30 s were performed. Intensity size distributions were obtained using the autocorrelation function.

### SPR

Antigen binding was evaluated by SPR using a Biacore 3000 instrument with CM-5 sensor chips that were activated with *N*-hydroxysuccinimide/*N*-ethyl-*N*′(dimethyl-aminopropyl) (NHS-EDC). For antigen immobilization, 0.15 mg/ml of α-syn(A53T) peptide (Genway) in 10 mM sodium acetate buffer, pH 4.0 was coupled to CM5 chips using NHS-EDC chemistry to a level of ~1500 RU. Serial dilutions of intrabodies (50–10,000 nM) in HBS-EP buffer (GE Healthcare Life Sciences) were injected at 30 μl/min for 2 min and 25 °C. Flow cells were regenerated by injection of 10 mM glycine (pH 4.0) followed by thorough washing with the running buffer. Additionally, a blank cell on the same sensor chip was used as a reference to correct for non-specific binding. Zero-concentration samples (blanks) were also included for double referencing. BIAevaluation software (GE Healthcare) was used to calculate values for *k*_on_, *k*_off_, and *K*_d_ by curve-fitting the data to a 1:1 Langmuir binding model using a simultaneous non-linear program. For comparison, the data were also evaluated by fitting the equilibrium binding responses to obtain *K*_d_ values using Prism 7 software.

### Models for V_H_NAC1 and the NAC region of α-syn

The starting structures for V_H_NAC1 were generated using the homology model engine of Swiss-Model^53^ and was based on the solved crystal structure for an anti-β2 adrenergic receptor nanobody (PDB: 3P0G)^54^. The homology model was generated for 121 out of 122 amino acid residues of V_H_NAC1 (Q residue at N-terminus was not present in the template structure). All simulations employed GROMACS^56^ using the CHARMM27 force field with CMAP and the OBC implicit solvent model^57^. The SD integrator of the GROMACS package was used with a timestep of 2 fs. The LINCS algorithm in GROMACS was used to constrain the length of all bonds^73^. The inverse friction constant of the SD integration was set to 91 ps^−1^. All interactions were run with without cutoffs. The homology model was subjected to a short steepest descent energy minimization followed by a short equilibration at a constant temperature of 300 K. For the REMD simulations, all residues except those in the hypervariable loops H1, H2, H3 and H4 were backbone-restrained^58,59^. The C_α_, N and C backbone atoms that do not belong to the hypervariable loops were restrained with the harmonic force constant of 2000 kJ/nm, while backbone atoms in H1-H4 along with side chains from all the residues were free to move. These restraints were imposed to simulate the limited motion of these residues in the folded domain. A total of 24 replicas at temperatures ranging from 300 K to 900 K were simulated for 20 ns (per replica) for the REMD simulations with an exchange attempt every 1 ps (500 steps). The g_cluster program in GROMACS with the linkage algorithm was used to cluster structures at the lowest temperature replica (300 K) to obtain the top 8 conformations. These conformations were used to start a new REMD, and simulated for another 8 ns to obtain the top 2 replicas in a similar manner.

In general, two models were considered for the NAC region: (i) a 19-residue α-helical peptide based on PDB: 1XQ8^61^; and (ii) a 13-residue random coil peptide generated by piecing together amino acids one-by-one. However, peptides of different lengths were used for modeling at different stages. For example, a 19-residue peptide (amino acids 61–79) was initially employed for docking calculations, but after the top complexes were obtained for the 19-residue peptide, the peptide was extended to 24 residues (amino acids 57–80) or to 27 residues (amino acids 57–83).

### Model for V_H_NAC1-NAC complex

The two conformations of V_H_NAC1 (N and ST) and 2 models of NAC (α-helical and random-coil) were used as starting structures to generate docked structures using the ClusPro docking software^62–65^. A total of 41 structures were generated (4 possible permutations and ~10 structures per V_H_NAC1-NAC pair). To rank these structures from the most stable to the least stable, all structures were energy minimized and equilibrated for 5 ns in the NVT ensemble at 300 K. Only the complexes that were stable in the equilibration runs were retained for further simulations. For each V_H_NAC1-NAC pair, a separate REMD was conducted. REMD was used as a technique to rank the complexes from the most stable (residing at the lowest temperature box 300 K; also temperature of interest) to the least stable (highest temperature 450 K). Several stable structures were obtained for the α-helical NAC peptide with either the N or ST conformations of V_H_NAC1; however, none of the complexes generated for the random coil model from ClusPro were stable in the equilibration simulations. To generate candidate structures involving the random coil peptide, the 20 complexes obtained for the α-helical NAC peptide with either the N or ST conformations of V_H_NAC1 were equilibrated at high temperature (550 K). The V_H_NAC1 was position restrained in the simulations to maintain its native structure while the α-helical NAC peptide was equilibrated to a higher temperature (to relax to a random coil conformation). The conformations thus obtained were equilibrated at 300 K for 5 ns followed by REMD simulations. The conformations sampled at 300 K were obtained for each case.

### Estimation of binding energy and PMF

To obtain the energy terms of interest, energy-groups were defined within GROMACS to calculate the energy of interaction between (i) V_H_NAC1 and the NAC peptide antigen and (ii) residues on V_H_NAC1 and the antigen. The energy of V_H_NAC1-NAC interaction for the top 5 complexes was determined. Umbrella sampling (US) simulations were used to obtain PMF of binding as a function of the radial distance between V_H_NAC1 and the NAC peptide antigen. US simulations were used to compare the change in PMF between the bound (NAC peptide in complex with V_H_NAC1) and unbound state (NAC peptide far away from V_H_NAC1). PMFs were compared for complexes with the same starting models for V_H_NAC1 and NAC peptide. Our PMF calculation is intended to describe the free energy changes associated with a transition between two “basins” in conformation space, where such basins may only represent metastable states (local mimima) as opposed to thermodynamic states. This is particularly relevant for the initial complex state for which we make specific choices. We use the US method to probe conformations along an order parameter that gradually moves the system from the bound basin to the unbound basin (in our case the order parameter is the distance between antibody and antigen). In US, molecules are unconstrained and free to sample all degrees of freedom “orthogonal” to the main order parameter. The initial configurations for the US windows were obtained by using the Pulling code in GROMACS. A total of ~80–96 windows were placed at a distance of 0.03 nm for windows at small separations (<2.5 nm) to 0.10 nm for windows at large separations (>4 nm) between V_H_NAC1 and the NAC peptide. Each window was run for 5–15 ns depending on the time it took for the free energy profiles to converge within each window. Overall, the simulations were run for a cumulative time of 500 ns to 1.5 μs. The g_wham program was used to obtain the PMF as a function of distance using WHAM, which estimates the statistical uncertainty of the unbiased probability distribution given the umbrella histograms, and subsequently computes the PMF that corresponds to the smallest uncertainty^74^.

### Calculation of electrostatic free energy of binding

DelPhi calculates electrostatic potentials in and around macromolecules and the corresponding electrostatic energies^67–69^. It incorporates the effects of ionic strength mediated screening by evaluating the PBEQ at a finite number of points within a three-dimensional grid box. The electrostatic free energy of binding is calculated as:

$${\rm{\Delta }}{\rm{\Delta }}{G}_{el}={\rm{\Delta }}{G}_{el}^{Complex}-{\rm{\Delta }}{G}_{el}^{vHH}-{\rm{\Delta }}{G}_{el}^{Ag}$$

For all our calculations using DelPhi, we used the following inputs according to Lippow *et al*.^35^: (i) PBEQ was solved for the complex (and not the encounter complex), this method has been previously implemented with success; (ii) PARSE parameters were used for partial atomic charges and radii^75^; and (iii) a dielectric constant of 4 was used for protein and explicit water, and 80 for implicit solvent regions; ionic strength was set at 0 M (no salt) and 0.15 M (with salt), and modeled with a 2.0-Å Stern Layer and a molecular surface generated with a 1.4-Å probe sphere. The number of grid points per angstrom (gpa) was set to 1.5. For all calculations, electrostatic free energy was calculated for 50–100 structures and the estimated value was obtained by averaging over them.

## Electronic supplementary material

Supplementary Information
