# Supplementary material for: Computational affinity maturation of camelid single-domain intrabodies against the nonamyloid component of alpha-synuclein
Source: Sci Rep. 2018 Dec 4;8:17611. doi: 10.1038/s41598-018-35464-7 (PMC6279780; doi:10.1038/s41598-018-35464-7)
Supplement: Supplementary file 1 — Supplementary Information [file 41598_2018_35464_MOESM1_ESM.pdf]

Supplementary Information File

Computational affinity maturation of camelid single-domain intrabodies against the nonamyloid component of alpha-synuclein

Sai Pooja Mahajan, Bunyarit Meksiriporn, Dujduan Waraho-Zhmayev, Kevin B. Weyant, Ilkay Kocer, David C. Butler, Anne Messer, Fernando A. Escobedo and Matthew P. DeLisa

a

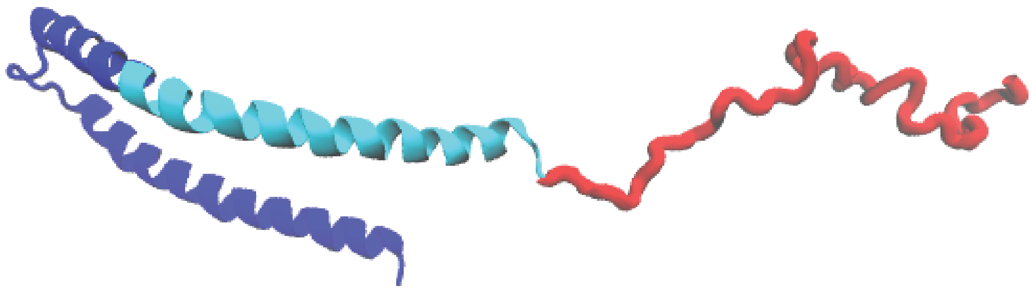

human α-Syn 1-140

1 MDVFMKGLSK AKEGVVAAAE KTKQGVAEAA GKTKEGVLYV GSKTKEGVVH GV**A**TVAEKTK

61 **EQVTNVGGAV VTGVTAVAQK TVEGAGSIAA ATGFV**KKDQL GKNEEGAPQE GILEDMPVDP

121 DNEAYEMPSE EGYQDYEPEA

b

NAC region 61-95

EQVTNVGGAVVTGVTAVAQKTVEGAGSIAAATGFV

Alpaca immunization peptide 53-87

ATVAEKTK**EQVTNVGGAVVTGVTAVAQKTVEGAGS**

13-residue peptide 66-78

VGGAVVTGVTAVA

19-residue peptide 61-79

EQVTNVGGAVVTGVTAVAQ

24-residue peptide 57-80

EKTK**EQVTNVGGAVVTGVTAVAQK**

Supplementary Figure 1. α-Syn protein and derivatives. (a) Representative structure of human α-syn, a 140-residue intrinsically disordered protein of unknown function that adopts many conformational forms upon interacting. (b) Amino acid sequences of human α-syn and the various NAC peptides that were used for the computational studies. Red bolded font corresponds to the nonamyloid component (NAC) region. Arrow denotes residue A53; a substitution of this residue with T results in a highly toxic version of α-syn that was used for genetic selection studies.

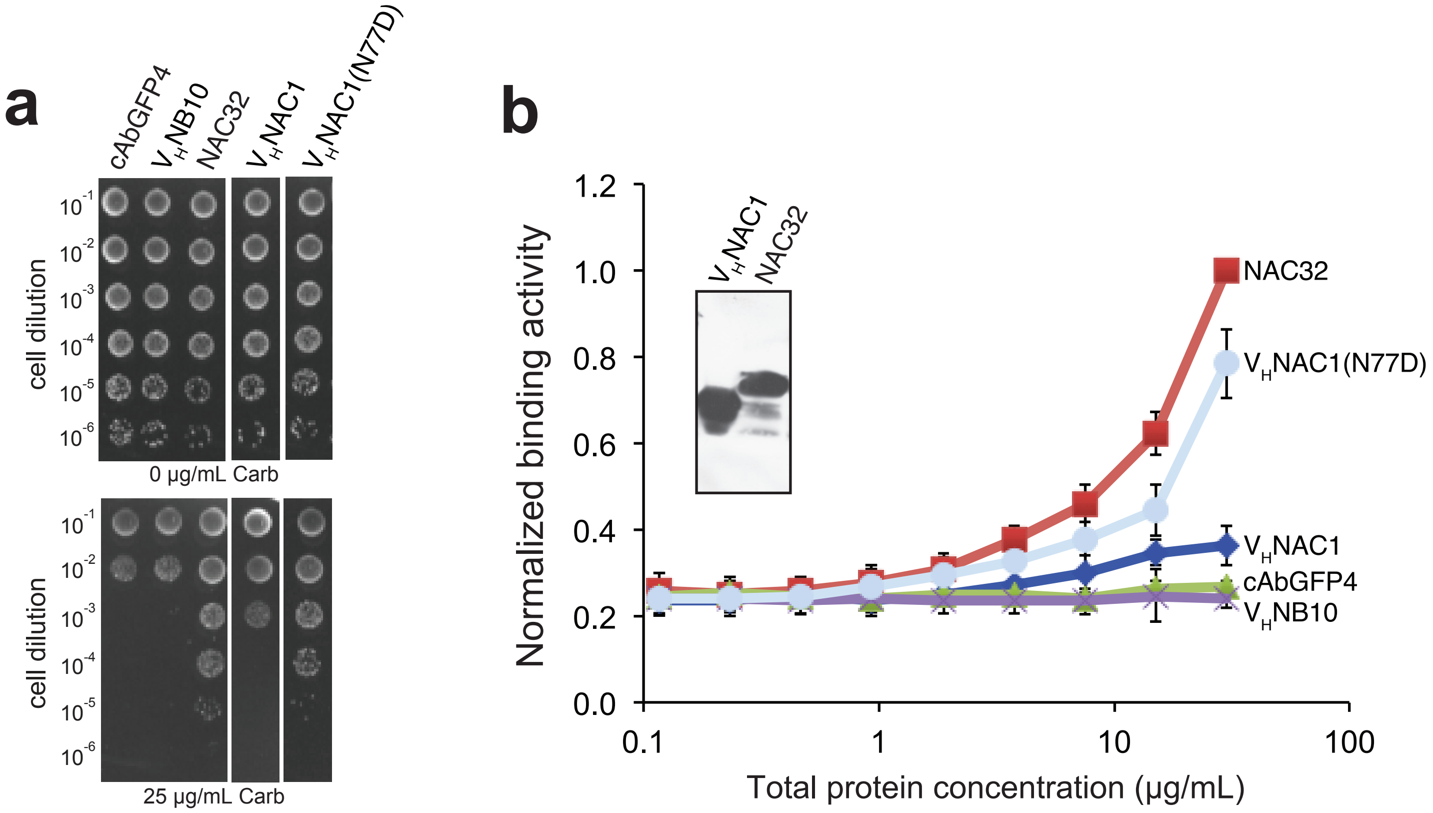

Supplementary Figure 2. Selection and characterization of NAC-specific VHH intrabodies. (a) Selective plating of *E. coli* MC4100 cells co-expressing α-syn(A53T)-Bla along with spTorA fusions to one of the following synthetic binding proteins: cAbGFP4, V_HNB10, NAC32, V_HNAC1, or V_HNAC1(N77D). Overnight cultures were serially diluted in liquid LB and plated on LB agar supplemented with Carb. Representative spot titers using 0 and 25 µg/mL Carb are shown here, the latter of which was the concentration used to select clone V_HNAC1 from the immune-focused VHH library. (b) Antigen binding activity of the same synthetic binding proteins in (a) as determined by ELISA. Each binding protein was expressed in *E. coli* BL21(DE3) cells from plasmid pET-21a without the spTorA signal peptide and with a C-terminal FLAG epitope tag. Binding activity in soluble cell lysates was measured by ELISA with microtiter plates coated with α-syn(A53T) as antigen. ELISA data are expressed as the mean ± standard deviation of three biological replicates. Inset shows Western blot analysis of V_HNAC1 and NAC32 expression level in soluble cell lysate fraction. Blot was probed with anti-FLAG antibody (see Supplementary Fig. 7 for uncropped version of blot).

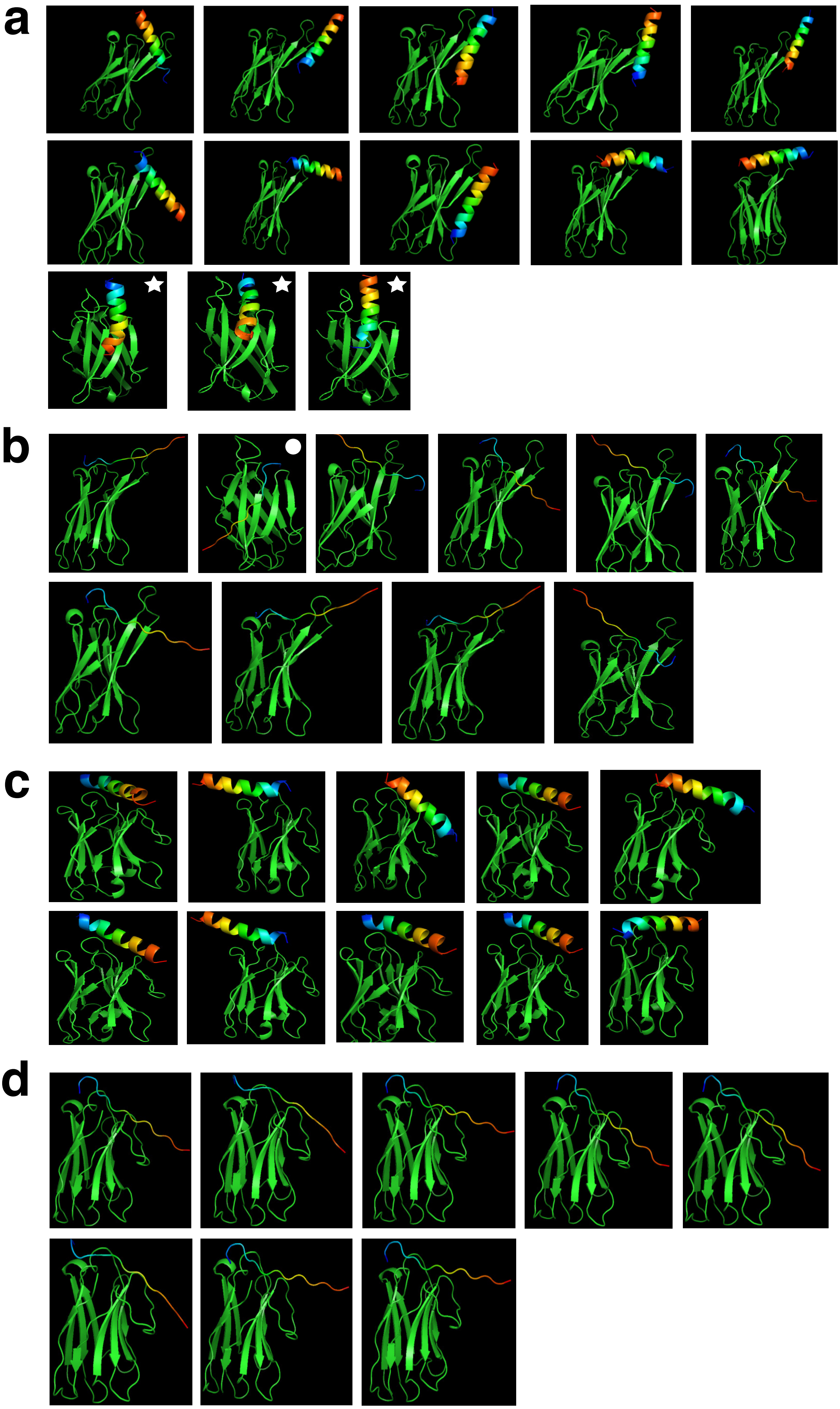

Supplementary Figure 3. Models of the V_HNAC1-NAC complex. Initial structures against the 19-residue α-helical peptide and 13-residue random coil NAC peptide were obtained from four docking runs in ClusPro: (a) V_HNAC1 (N conformation) with 19-mer α-helical peptide (3 structures marked with white star were generated by manually docking the NAC peptide in a potential binding site formed by the H1, H2, and H3 loops not identified in the ClusPro runs for the α-helical peptide but identified in ClusPro runs for the random coil peptide indicated by white circle); (b) V_HNAC1 (N conformation) with 13-mer random coil peptide; (c) V_HNAC1 (ST conformation) with 19-mer α-helical peptide; (d) V_HNAC1 (ST conformation) with 13-mer random coil peptide. All structures were energy minimized and equilibrated at 300K using MD simulations. NAC peptides are shown in rainbow coloring, from blue (N-terminus) to red (C-terminus).

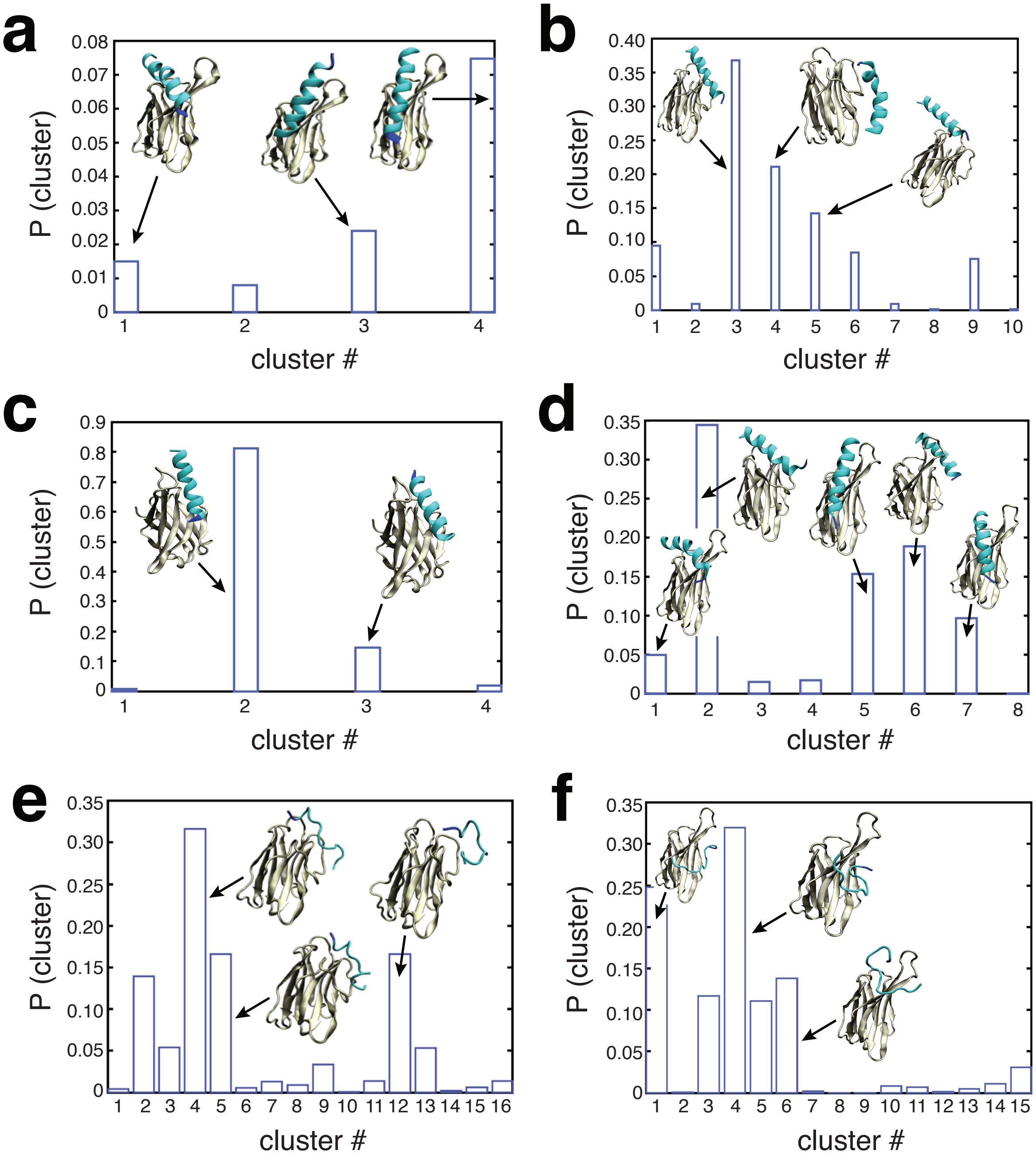

Supplementary Figure 4. Additional models of V_HNAC1 in complex with NAC peptide Probability distribution of structures obtained from REMD simulations for: (a) V_HNAC1 (N conformation) with 19-mer α-helical peptide; (b) V_HNAC1 (ST conformation) with 19-mer α-helical peptide; (c) V_HNAC1 (N conformation) with 19-mer for manually generated binding site shown in Supplementary Fig. 3a; (d) combined simulations with top conformations obtained from (a), (b) and (c); (e) V_HNAC1 (ST conformation) with 13-mer random coil peptide; and (f) V_HNAC1 (N conformation) with 13-mer random coil peptide. The N-terminus of the NAC peptide is shown in cyan.

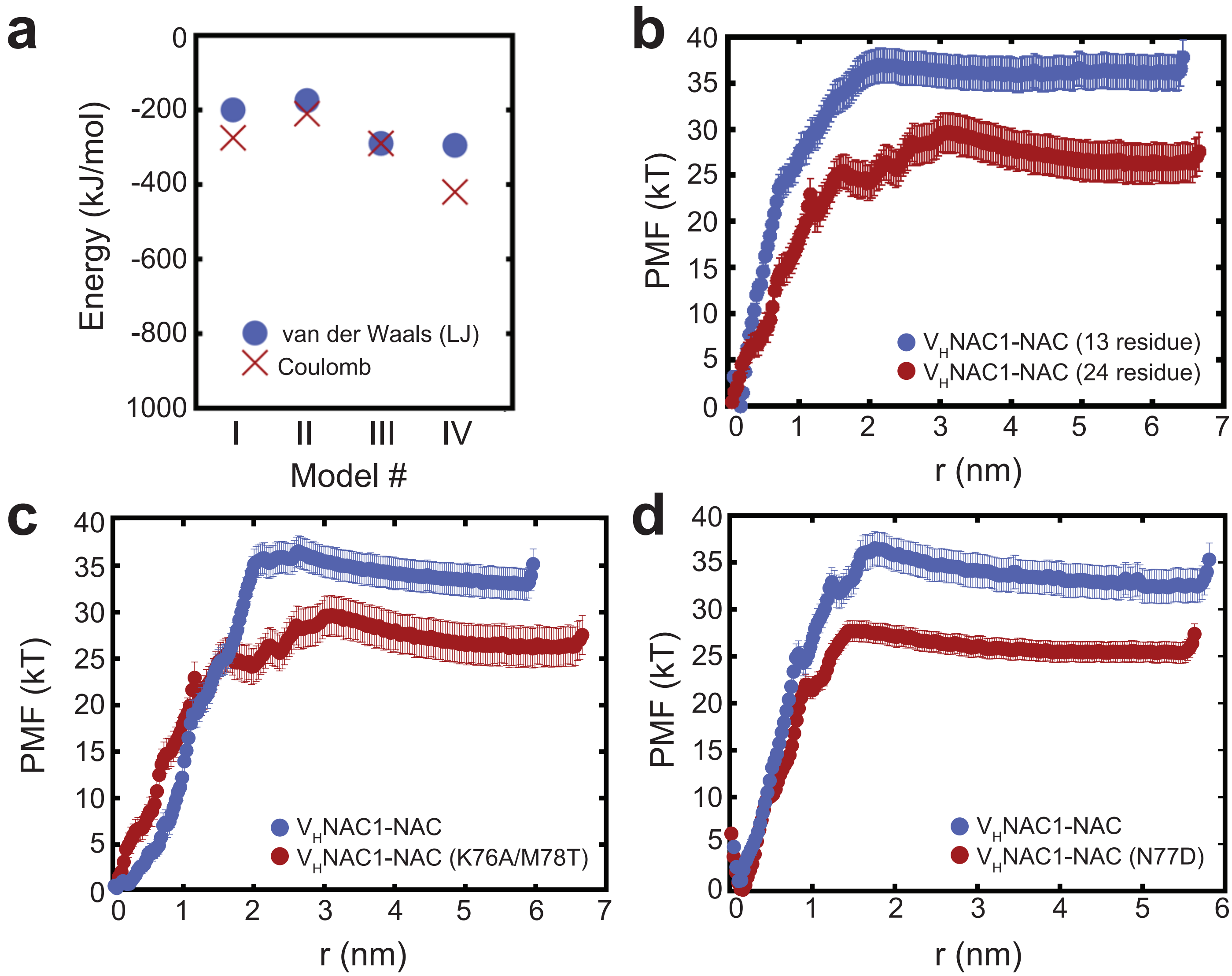

Supplementary Figure 5. Characterization of different V_HNAC1-NAC models. (a) Energy of interaction calculated separately for the van der Waals type interactions and Coulombic interactions. Comparing strength of binding using US simulations to determine PMF for: (b) V_HNAC1 with 13-mer or 24-mer NAC peptides for Model III; (c) V_HNAC1 or K76A/M78T mutant with 24-mer NAC peptide for Model III; (d) V_HNAC1 or N77D mutant with 24 NAC peptide for Model V.

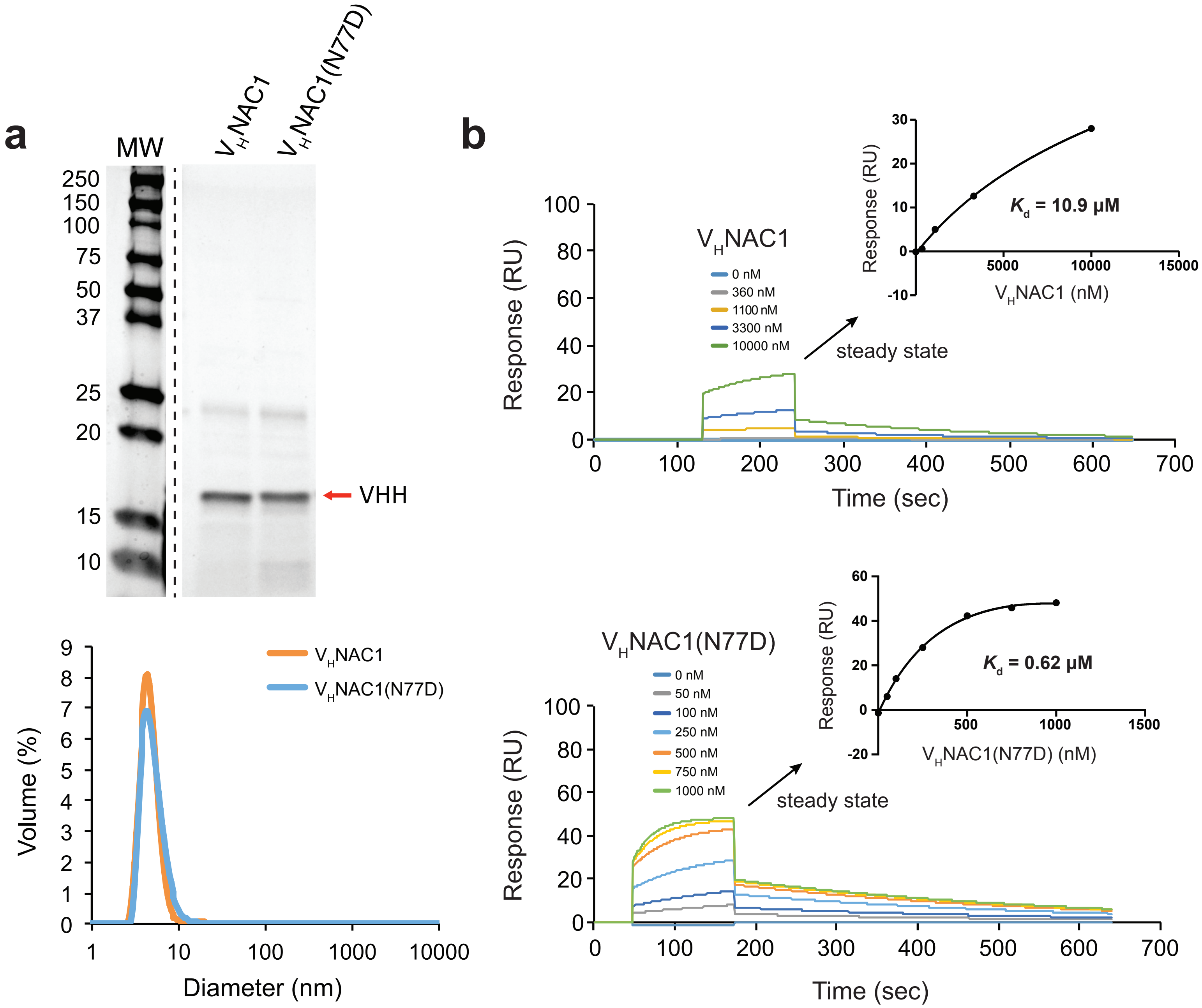

Supplementary Figure 6. Binding affinity of VHH proteins. (a) SDS-PAGE (upper panel) and DLS (lower panel) analysis of purified VHH clones prior to SPR analysis. Each VHH was expressed from pET-21a in BL21(DE3) cells and purified from soluble cell lysates using standard Ni-NTA chromatography followed by size exclusion chromatography (SEC). An equivalent amount of each purified VHH protein was loaded into SDS-PAGE gel followed by electrophoretic separation and visualization by Coomassie brilliant blue staining. Dashed line indicates where this gel image was spliced to remove empty lanes/irrelevant samples. For analysis of protein aggregation using DLS, purified VHH proteins were concentrated to >0.1 mg/ml and 400 µl was added to disposable plastic cuvettes for DLS measurements using a Malvern Zetasizer Nano ZS For each sample, quadruplicate measurements consisting of 20 runs of 30s were performed. Results shown here are representative of the data collected. (b) The binding kinetics of V_HNAC1 and N77D mutant were monitored using Biacore. Purified α-syn(A53T) was immobilized at low concentrations and the response of different concentrations of each VHH as indicated was compared with an empty flow cell. Three independent experiments were carried out for each combination. Representative results are depicted. The global fit was performed by fitting the equilibrium binding responses to a 1:1 Langmuir binding model using a simultaneous non-linear program using BIAevaluation software, which was used to calculate values for k_{on}, k_{off}, and K_d, which are given in Table 1 (see Supplementary Fig. 8 for curve fit of the data in panels (b). For comparison, the data were also evaluated by fitting the equilibrium binding responses to obtain K_d values as shown in the insets.

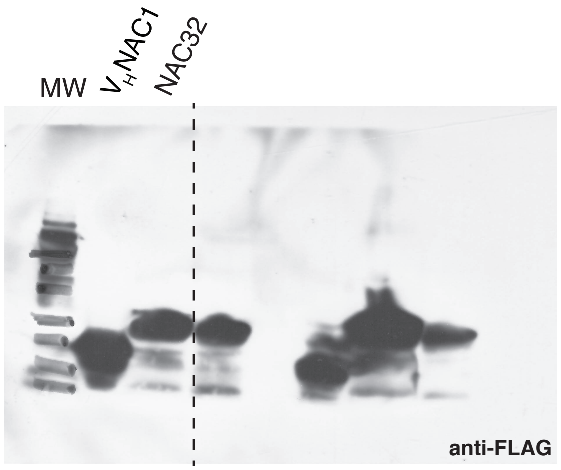

Supplementary Figure 7. Uncropped image corresponding to Supplementary Figure 2 inset. Samples to the right of the dashed line are irrelevant. MW = molecular weight ladder. Detection with anti-FLAG antibody.

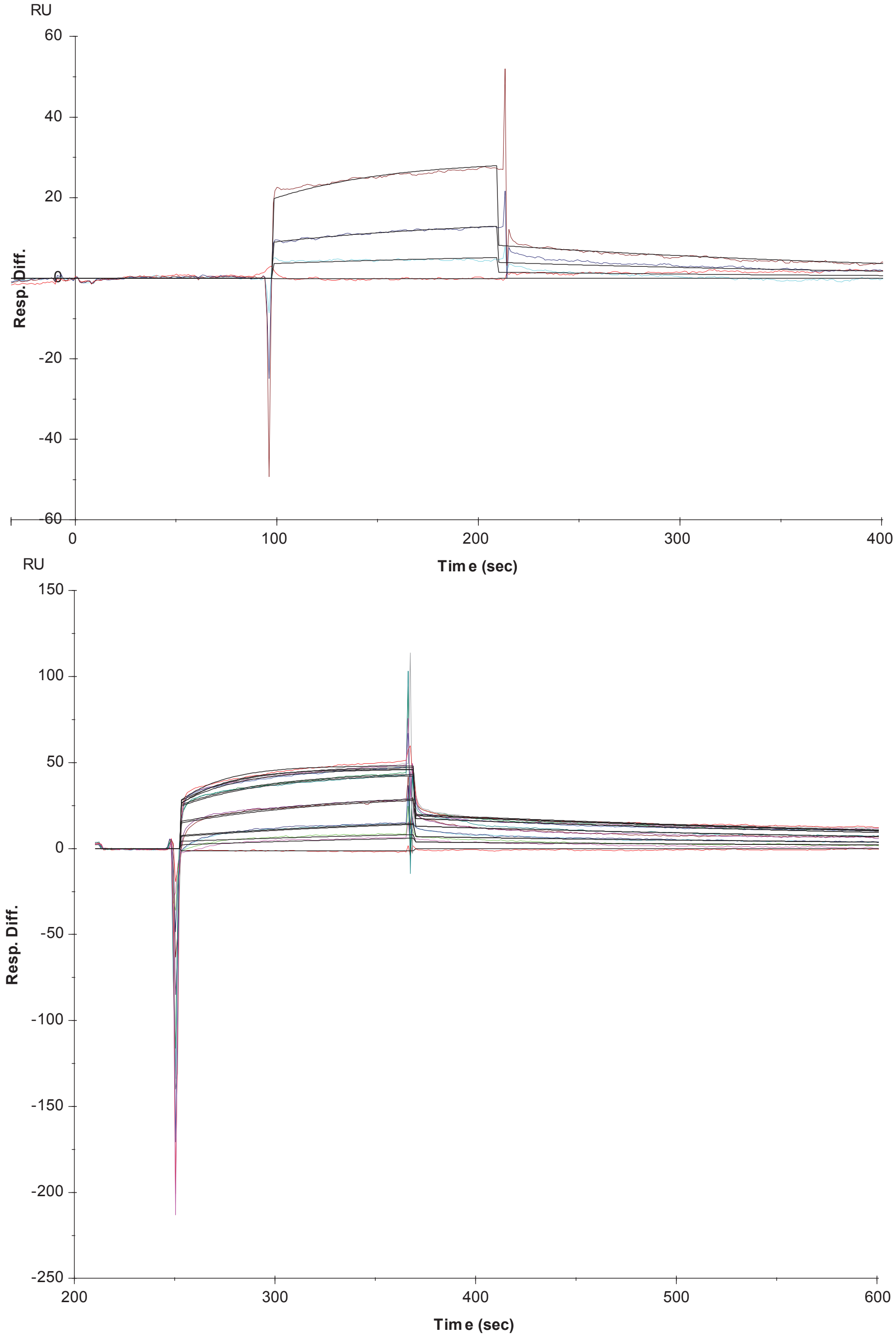

Supplementary Figure 8. Curve fit of the data to a 1:1 Langmuir binding model using a simultaneous non-linear program using BIAevaluation software, which was used to calculate values for k_{on}, k_{off}, and K_d (see Table 1).
